# The multidimensional attractiveness of marathon events: how self-efficacy bridges perception to participation

**DOI:** 10.3389/fpsyg.2025.1649195

**Published:** 2025-08-21

**Authors:** Mingtao Wang, Mengkai Wang, Xinyue Yuan, Xi Zhao, Yongtao Zhang

**Affiliations:** School of Economics and Management, Chengdu Sport University, Chengdu, China

**Keywords:** perceived attractiveness, marathon participation, self-efficacy, mixed-methods research, behavioral intention

## Abstract

**Introduction:**

Although marathon events have gained global popularity and align closely with public health objectives, limited research has explored how individuals perceive the attractiveness of these events and how such perceptions influence participation intention. This study introduces the concept of “perceived attractiveness” as a multidimensional evaluation encompassing emotional, symbolic, cultural, and convenience facets related to participation. Drawing upon self-determination theory and self-efficacy theory, the study investigates how perceived attractiveness predicts participation intention, mediated by exercise-related self-efficacy.

**Methods:**

A sequential exploratory mixed-methods approach is adopted. Initially, grounded theory is applied to analyze 45 semi-structured interviews with marathon participants, yielding four dimensions of perceived attractiveness: psychological engagement, symbolic and social value, cultural and environmental atmosphere, and participation convenience. These dimensions inform the development of a structured questionnaire, which is subsequently administered to 426 respondents across China. Structural equation modeling (SEM) is employed to examine the hypothesized relationships among the variables.

**Results:**

All four dimensions of perceived attractiveness significantly predict marathon participation intention. Perceived symbolic and social value exerts the strongest effect, followed by participation convenience, cultural and environmental atmosphere, and psychological engagement. Self-efficacy is found to partially mediate all relationships, indicating that attractive perceptions enhance confidence, which in turn promotes participation intention.

**Discussion:**

The findings suggest that individuals are motivated to participate in marathons not only by intrinsic goals but also by their perceptions of the event’s external characteristics. Perceived attractiveness acts as a cognitive-affective precursor to action, especially when mediated by self-efficacy. These insights contribute to sport psychology literature and offer practical implications for event organizers, educators, and policymakers seeking to foster sustained engagement in physical activity.

## 1 Introduction

Promoting mass participation in physical activity has become a global public health priority, recognized not only for its physical health benefits but also for enhancing psychological wellbeing and community engagement (World Health Organization, 2022). Marathon running, in particular, has emerged as an inclusive form of physical activity that appeals to a wide demographic, uniting personal health goals and collective identity (Scheerder et al., 2015). According to a report by RunRepeat (2023), approximately 1.1 million runners complete a marathon each year. Unlike elite-status athletic competitions, marathons are accessible to both amateur and professional participants, offering an experiential platform grounded in personal achievement rather than commercial spectacle. In China, this trend aligns with the national “Healthy China” initiative, which has catalyzed rapid growth in road-running events. According to the 2023 China Road Running Races Blue Book, the country hosted 622 marathons and half-marathons, attracting over 6 million participants, with some weekends registering more than 400,000 runners. Despite this surge in public enthusiasm, the rate of sustained participation remains unclear. It is still not well understood which specific attributes of marathons are perceived as sufficiently attractive to influence the decision to participate.

Existing literature has primarily emphasized frameworks centered on infrastructure and demographic factors (Downward et al., 2014), and has extensively explored the motivations behind marathon participation. However, these motivational studies often interpret behavior based on individuals who already exhibit participation tendencies, focusing mainly on internal drivers of action (Larumbe-Zabalamt et al., 2020; León-Guereño et al., 2020a; Bizen and Ninomiya, 2022). They tend to overlook how individuals evaluate the event itself—specifically, how people perceive the appeal of a given sport. The notion of attractiveness is typically defined as a positive evaluation of the qualities or potential benefits of an object that motivates individuals to interact with it (Harris et al., 2003; Ellegaard and Ritter, 2007). While attractiveness has been examined in athlete-centered contexts—focusing on physical appeal, fame, or likability (Lee and Park, 2014; Mutz and Meier, 2016; Frank and Mitsumoto, 2023)—it has not yet been systematically theorized in the domain of mass-participation sport events such as marathons. Given the high demands marathons place on time, training, and psychological commitment, understanding perceived event attractiveness is crucial. We define perceived marathon attractiveness as the subjective evaluation of event features. Such perception serves as a psychological precursor to behavioral intention, potentially triggering motivation, enhancing self-efficacy, and ultimately influencing one’s willingness to participate.

To explain how perceived attractiveness affects behavioral intention, this study draws upon motivational and self-efficacy theories (Deci and Ryan, 2000). Subjective evaluations of external contexts are believed to stimulate internal motivation and self-belief, thereby increasing one’s intention to act. This has been widely supported in physical activity research, where self-efficacy has consistently emerged as a key psychological predictor of both the initiation and maintenance of exercise behavior (Bauman et al., 2012; Rhodes et al., 2017). Accordingly, in this study, perceived attractiveness is not only conceptualized as a cognitive-affective evaluation of marathon events but also as an antecedent condition that fosters self-efficacy and, in turn, predicts participation intention. Particularly in the context of marathons—where physical preparation and psychological commitment are both demanding—the interplay between attractiveness and self-efficacy provides a compelling theoretical lens through which to understand sustained public engagement in long-distance running (McCormick et al., 2015).

We employed a sequential exploratory mixed-methods design. This involved grounded theory analysis of 45 in-depth interviews, followed by structural equation modeling using survey data from 426 participants. This methodological approach aligns with recent calls for methodological diversification in sports management research (Bakhsh et al., 2024), allowing for the generation of theory through qualitative exploration and its empirical validation through quantitative analysis.

This study addresses three core research questions: (1) what are the key dimensions that constitute the perceived attractiveness of marathon events? (2) How do these dimensions influence individuals’ intentions to participate? (3) What evidence-based strategies can guide policymakers and event organizers in promoting sustainable engagement in public running programs? By answering these questions, this research aims to advance theoretical understanding of voluntary sports participation and inform psychologically grounded interventions that support broader and more sustained public involvement in marathon running and health promotion.

## 2 Literature review

### 2.1 The rise of marathon running and its societal significance

Over recent decades, marathon running has undergone a profound transformation—from an elite athletic competition to a globally embraced form of mass participation sport. Once associated primarily with high-performance endurance athletes, marathons are now widely recognized as symbolic and inclusive public events that embody values such as perseverance, self-discipline, and personal achievement (Scheerder et al., 2015). This shift reflects a broader reconfiguration of sport and physical activity in contemporary societies, where health promotion, lifestyle enhancement, and civic identity have become elements of public discourse surrounding exercise (Oldridge-Turner et al., 2022).

In China, marathon running has rapidly emerged as one of the most visible and accessible forms of urban physical activity. Driven by national fitness initiatives and urban development agendas, the number of officially certified marathon events increased from just 22 in 2011 to over 1800 by 2019, before experiencing a temporary decline due to the COVID-19 pandemic (Zheng et al., 2023). According to the 2023 China Road Running Events Blue Book, more than 622 races were held across the country in 2023, attracting over 6 million participants across various distance categories—including full marathons, half marathons, and mini runs (CAA, 2024). These events serve not only athletic and recreational purposes but also function as platforms for city branding, health advocacy, and grassroots social mobilization (Broudehoux, 2017).

Unlike traditional sports confined to competitive settings or specialized facilities, marathon events are typically held in open urban spaces and attract heterogeneous groups of participants from diverse social backgrounds. This inclusivity enhances their symbolic appeal as public rituals of endurance, solidarity, and self-transcendence (Bale, 2004; Lassalle et al., 2019). Furthermore, the growing visibility of marathons through digital platforms and media coverage has elevated their cultural significance, contributing to the normalization of long-distance running as an aspirational and identity-forming activity (Shipway and Jones, 2007). As a result, marathon running has evolved into a hybrid phenomenon—simultaneously fulfilling individual health goals, facilitating social interaction, and satisfying deeper psychological needs for recognition and meaning (Waśkiewicz et al., 2018).

Despite their widespread popularity, marathon events differ significantly from other forms of recreational physical activity in terms of preparation requirements, time investment, and psychological commitment (Hamstra-Wright et al., 2013). The decision to register and train for a marathon often involves months of personal planning, perceived self-efficacy, and motivational resilience. These behavioral and emotional demands distinguish marathon running from more spontaneous leisure-time exercise, suggesting the need to reconceptualize marathon participation as a complex psychosocial process rather than a simple behavioral choice (León-Guereño et al., 2020b).

Accordingly, understanding why individuals choose to engage in marathon events requires a multi-dimensional approach that considers not only structural facilitators (e.g., infrastructure, accessibility) but also subjective psychological drivers. In this regard, the perceived attractiveness of marathons—including their symbolic significance, perceived value, and experiential rewards—may play a critical role in shaping participation decisions. However, few studies have systematically examined how these perceptual dimensions are structured or how they influence individuals’ willingness to participate in the context of endurance-based, mass-participation events, such as marathons.

### 2.2 Behavioral characteristics and participation patterns in marathon events

In contemporary sport psychology research, a clear conceptual distinction is drawn between the internal motivations that drive individual behavior and the external attributes that render a sport activity attractive (Duda, 2005; Clancy et al., 2016). This distinction is particularly salient in the context of marathon running—a voluntary, endurance-based form of physical activity that is often self-initiated and sustained over long periods (Gordon et al., 2017).

Motivation refers to the internal psychological needs, goals, and drives that energize individuals to engage in and persist with specific behaviors (Lai, 2011; Peters, 2015). In the context of marathon participation, such motives may include the pursuit of health and fitness, psychological wellbeing, emotional catharsis, social connection, or personal challenge (Waśkiewicz et al., 2018; Waśkiewicz et al., 2019). These motivational dimensions have been extensively studied using validated instruments such as the Motivations of Marathoners Scale (MOMS) (Zach et al., 2017), which identifies constructs such as achievement, coping, self-esteem, and affiliation.

Attractiveness is not synonymous with motivation (Vogel et al., 2010). Instead, it is a perceived property of the activity itself—the extent to which the characteristics of a sport are seen as compelling, engaging, or personally valuable. Attractiveness is shaped by how individuals interpret and evaluate various facets of the sport, including its symbolic meaning, emotional tone, community culture, perceived benefits, and experiential outcomes (Kim and Trail, 2010).

In this regard, motivation originates from within the individual, whereas attractiveness resides in the activity as it is subjectively perceived. Well-established theoretical frameworks in sport behavior and leisure studies support this conceptual separation. For instance, Iso-Ahola (1980) model of leisure motivation posits that behavior results from the dynamic interaction between internal “pushes” and external “pulls”—a logic further refined in dual-process models of exercise behavior (Chatzisarantis et al., 2016). Within such models, perceived attractiveness functions as an environmental cue that activates or inhibits latent motivational tendencies. For example, an individual with a dormant desire for self-transcendence may not act on that desire unless a sport is perceived to embody that symbolic potential (Brewer and Howarth, 2012).

Recent research supports this differentiation in the context of endurance sports (Tammelin et al., 2003; Scheer, 2019). Studies have shown that perceptions of a sport’s emotional rewards, identity signaling, and community affiliation can significantly influence willingness to participate, even after controlling for baseline motivation (Readdy et al., 2014). Marathon running, in particular, is increasingly conceptualized not just as a physically demanding activity, but as a multi-dimensional social and emotional experience with symbolic, ritualistic, and affective dimensions (Robinson et al., 2014).

Accordingly, this study does not aim to further dissect individual motivations per se, but rather to explore the perceptual dimensions of marathon attractiveness—that is, what makes marathon running appealing to the general public. By identifying and validating these perceived dimensions, this research provides insight into how the structure, culture, and symbolic features of marathon running align with or activate individual motivations, thereby influencing participation intention. This analytical shift from “why people run” to “what about running makes people want to run” offers a novel contribution to the sport participation literature.

## 3 Methods

Unlike purely qualitative or quantitative approaches, mixed methods research allows for a deeper and more comprehensive understanding of phenomena, offering more holistic solutions (Johnson and Onwuegbuzie, 2004; McKim, 2017). This study adopts an exploratory sequential mixed-methods design to examine the perceived dimensions of marathon attractiveness from participants’ perspectives and to assess their influence on willingness to participate. In light of the absence of a systematic definition or established theoretical framework for the core construct—“perceived attractiveness of marathon events”—the qualitative phase was prioritized. This inductive approach aimed to elicit rich, participant-driven accounts of what makes a marathon appealing and to identify its underlying dimensions. Drawing on grounded theory methodology (Glaser et al., 1968), we avoided imposing preconceptions and systematically developed the concept based directly on participant narratives. This approach ensured the emerging categories authentically reflected participants’ real-world perceptions, enhancing ecological validity and the authenticity of the findings. This strategy is consistent with best practices in exploratory mixed-methods research, where the qualitative phase provides the foundation for developing contextually grounded constructs that are subsequently operationalized and tested through quantitative methods (Creswell et al., 2003; Bakhsh et al., 2024).

**Figure 1:**
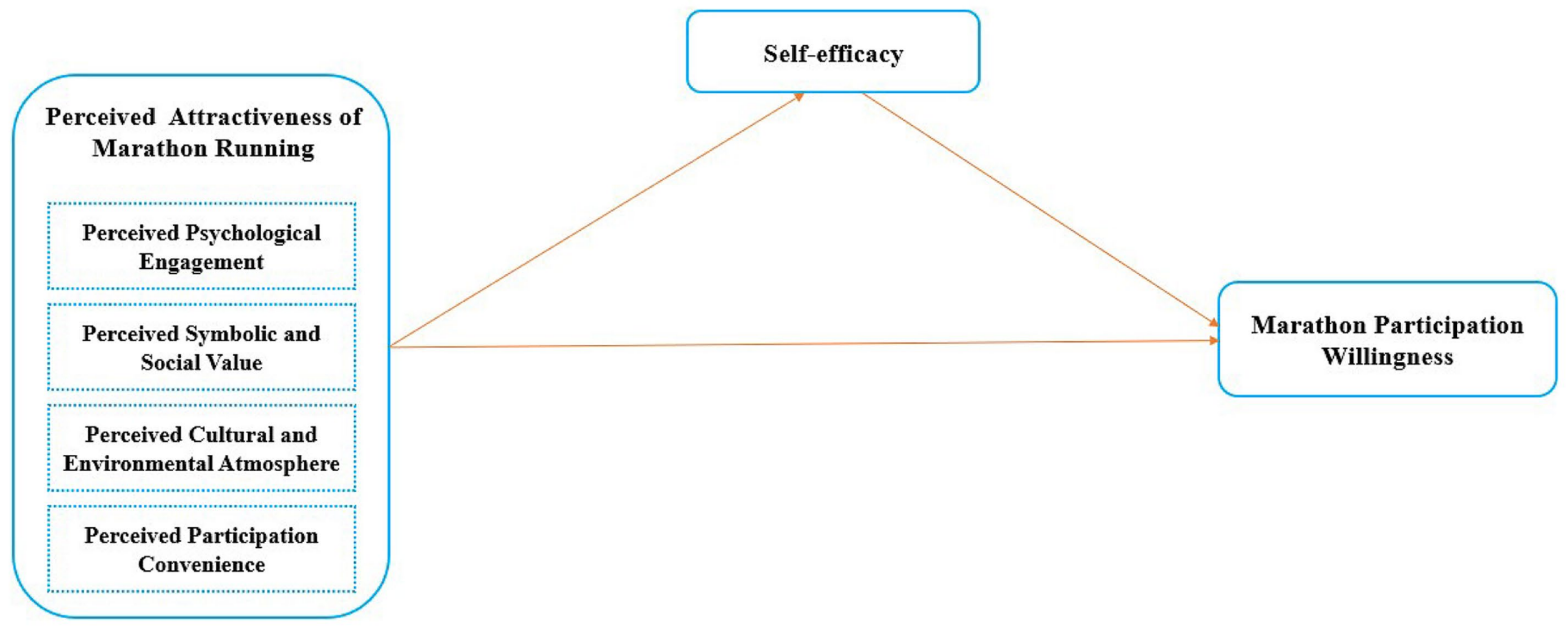
Research model.

Following Bakhsh et al. (2024) framework for high-quality mixed-methods research, this study adheres to four criteria: justification, type, distinct results, and mixing. This study adheres to these criteria through the following steps. First, justification: Previous studies on public sport participation have predominantly relied on singular qualitative or quantitative methods. While qualitative approaches risk overlooking generalizable patterns, purely quantitative methods may fail to capture emerging public perceptions (Johnson and Onwuegbuzie, 2004). By integrating grounded theory and structural equation modeling, this study combines the inductive depth of qualitative insights with the generalizability of quantitative testing, thereby enhancing both the credibility and comprehensiveness of the findings (Risjord et al., 2002; Hurmerinta-Peltomäki and Nummela, 2006). Furthermore, the unique context of marathon running as a psychologically and socially meaningful activity necessitates a flexible methodology to uncover novel dimensions of attractiveness. Second, type: This research adopts an exploratory sequential design (Creswell et al., 2003), comprising a qualitative phase involving grounded theory analysis of 45 in-depth interviews to identify perceptual dimensions of marathon attractiveness, followed by a quantitative phase administering a survey to 426 participants and analyzing data via structural equation modeling (SEM) to validate and quantify the impact of identified dimensions on participation willingness. This design prioritizes qualitative insights to inform the development of quantitative instruments, ensuring survey items align with participants’ lived experiences (Bakhsh et al., 2024). Finally, adhering to Bakhsh et al. (2024) third and fourth criteria (distinct results and mixing), the study separately discusses and integrates findings from qualitative and quantitative phases (Figure 1).

## 4 Qualitative method

### 4.1 Qualitative data collection

The qualitative phase of this study aimed to explore the perceived attractiveness of marathon running from the perspective of actual participants. To this end, a grounded theory approach was adopted, as introduced by Glaser et al. (1968). Grounded theory facilitates the systematic analysis of empirical data to inductively develop concepts and theoretical models that reflect the essence of the phenomenon under study (Holt et al., 2022). This method provides a scientific and rigorous framework by integrating empirical observation with theoretical abstraction.

Following ethical approval from the university’s Institutional Review Board, semi-structured in-depth interviews were conducted between September and November 2023. A standardized interview protocol (see Table 1) was developed, incorporating open-ended prompts designed to elicit participants’ experiences, impressions, and expectations regarding marathon participation. Interview topics included motivational triggers, emotional evaluations, perceived benefits, and environmental factors that contributed to participants’ perceptions of marathon running as an attractive form of physical activity.

**Table 1:** Semi-structured interview outline.

Serial number	Basic questions
1	We are researching to understand what makes marathon running appealing to participants. This interview will take approximately 30–60 min. Are you willing to participate?
2	How did you first come to know about marathon running, and what were your first impressions when you encountered it?
3	Before deciding to register for a marathon, what aspects of marathon running were you most interested in or focused on?
4	Please recall one of your marathon running experiences in detail. What aspects of the activity itself—such as the running process, personal feelings, or environmental features—did you find particularly impressive, memorable, or engaging?
5	Apart from the running itself, what other aspects related to the overall experience—such as atmosphere, community, event design, or cultural elements—made you feel exceptionally positive, immersed, or satisfied?
6	Based on your overall marathon experience, what would you say are the most unique or attractive features of marathon running compared to other types of sports or physical activities?
7	Were there any aspects of marathon running or the event organization that you felt could have been improved or that diminished your experience or motivation?
8	Given your past experiences, do you plan to participate in future marathons, and if so, why? What elements would influence your willingness to continue participating?

Participants were recruited through purposive sampling based on two inclusion criteria: (1) previous participation in at least one urban marathon (full, half, or mini); and (2) the ability to articulate personal experiences with clarity and depth. All participants were informed of the academic nature of the study and received guidance to facilitate meaningful engagement. To preserve thematic focus, interruptions during interviews were minimized, fostering a relaxed yet purposeful atmosphere conducive to open dialogue (Foley et al., 2021). In total, 45 interviews were completed, including 38 conducted in person and seven via secure video conferencing platforms. Each session lasted approximately 30–60 min. All interviews were audio-recorded, transcribed verbatim, and produced a corpus of over 110,000 words of textual data for analysis.

The sample included 20 female and 25 male participants, geographically distributed across China: 15 from the eastern region (e.g., Shanghai, Jiangsu), 15 from central provinces (e.g., Hunan, Henan), and 15 from the western region (e.g., Sichuan, Chongqing). This geographic and demographic diversity enhanced the conceptual variation of the sample by grounded theory principles, ensuring that the emerging themes captured a broad range of participant experiences and perspectives.

### 4.2 Qualitative data analysis

To ensure the standardization and rigor of the qualitative analysis, a structured data analysis protocol was implemented. An interview panel was formed, consisting of four members: two full professors and two PhD holders, each with extensive expertise in sports event research. Before the study commenced, the panel conducted a workshop to establish consensus among members regarding the definition and criteria for coding. Each member conducted individual in-depth interviews with the subjects and documented their respective coding processes and outcomes. Subsequently, upon completion of the coding phase, the interview panel members engaged in discussions, comparing their coding results, addressing and explaining disparities, and collaboratively devising a unified coding system. Finally, for concepts and categories presenting challenges in categorization, the interview panel members iteratively conducted coding, followed by analysis, comparison, and deliberation until a consensus was reached.

## 5 Qualitative results and discussion

### 5.1 Open coding

Open coding, the initial phase of grounded theory analysis, entails deconstructing interview transcripts into discrete conceptual units through line-by-line and paragraph-level interpretation (Glaser, 2016). In this study, all interview transcripts were systematically organized and reviewed. Non-substantive or repetitive content was excluded, and the remaining data were subjected to a detailed semantic analysis to identify salient perceptions related to marathon participation.

Through this process, line-by-line coding was applied to the transcripts to generate initial codes that captured meaningful concepts. A total of 52 initial concepts were identified during open coding. These were then constantly compared, refined, and grouped through an iterative coding process, resulting in 17 subcategories. Each subcategory represents a unique but interrelated conceptual element that reflects how individuals perceive the attractiveness of participating in a marathon event (see Table 2).

**Table 2:** Open coding example.

Interview content coding	Conceptualization	Categorization	Category properties	Dimensions of properties
Completing the entire marathon was one of the hardest things I’ve ever done I could feel my body breaking down after just 15 kilometers.…	Physical endurance demand Mental toughness requirement …	a1 perceived challenge intensity	Physical Demands	High/Low
Crossing that finish line gave me an overwhelming sense of personal fulfillment Even though it was exhausting, I genuinely enjoyed every moment of the race …	Emotional gratification Psychological satisfaction …	a2 emotional reward	Emotional Payoff	High/Low
Training for marathons has helped me grow both physically and mentally Running gave me the confidence to believe I can handle anything in life…	Self-development experience Psychological empowerment …	a3 personal growth	Self-Development	High/Low
I signed up with a clear goal: to prove something to myself Reaching my target time felt like a major life accomplishment …	Achievement-driven mindset Personal objective pursuit …	a4 goal orientation	Achievement Focus	High/Low
I was so into it that I did not even realize I had run 10 kilometers During the race, I entered a zone where nothing else mattered…	Deep task immersion Enjoyable performance focus …	a5 flow experience	Immersive State	Strengths/ Weaknesses
This marathon wasn’t just a race—it symbolized overcoming my personal struggles Running represents resilience and progress for me, not just sport…	Event symbolic value Personal life metaphor …	a6 symbolic meaning	Meaning-Making	Strengths/ Weaknesses
I ended up chatting with several strangers along the route—it felt natural The conversations during the run made me feel genuinely connected to others…	Peer bonding opportunity Interpersonal connection …	a7 social interaction	Peer Engagement	High/Low
Being a marathon runner is a big part of who I am now Running helps me express the real me in a way nothing else does …	Self-identity display Personal values projection …	a8 identity expression	Self-Projection	High/Low
There’s a strong sense of belonging at every marathon I’ve attended We cheer each other on, even if we have never met before…	Sense of belonging Group membership identity	a9 community connection	Social Bonding	Strengths/ Weaknesses
I loved how the local culture was incorporated into the event ceremony From the music to the food, everything reflected this city’s identity…	Cultural immersion context Event-local cultural fusion …	a10 cultural integration	Local Culture	Good/Bad
Running through scenic trails with mountains in the background was unforgettable The fresh air and natural surroundings made the run more enjoyable…	Scenic physical setting Natural surroundings enjoyment …	a11 natural environment	Scenic Setting	Good/Bad
The atmosphere was electric, and it kept my energy levels high You could feel the excitement from the crowd and the runners alike…	Event excitement vibe Stimulating competition mood …	a12 event atmosphere	Event Vibe	Good/Bad
The event layout was beautiful and made the entire venue feel world-class The route was well-designed and offered stunning views throughout …	Route visual appeal Spatial design quality …	a13 spatial aesthetics	Visual Appeal	Good/Bad
I appreciated being able to choose between different starting slots The event time was perfect—it did not interfere with my work schedule	Schedule convenience Temporal participation freedom …	a14 time flexibility	Schedule Ease	Good/Bad
The fee was reasonable considering the services and race pack we received For the price, the overall experience definitely exceeded my expectations	Financial affordability Participation value ratio …	a15 cost-efficiency	Affordable Access	Good/Bad
Toilets, water stations, everything was available and easy to access Staff were helpful and medical support was visible throughout. ….	On-site facility provision Organizational support quality …	a16 infrastructure support	Facility Quality	Good/Bad
The subway dropped us off just a short walk from the starting point They arranged shuttle buses that made commuting really easy …	Transportation convenience Location accessibility …	a17 traffic accessibility	Easy Access	Good/Bad

Open coding involves a significant amount of text analysis. In the interest of brevity, only a portion of the table is presented here.

### 5.2 Axial coding

Axial coding, the second stage of grounded theory analysis, involves the continuous examination, comparison, and synthesis of the relatively independent categories identified during open coding (Vollstedt and Rezat, 2019). This phase facilitates the extraction of overarching concepts and the formation of thematic structures. In this study, we re-examined the original data and comprehensively evaluated the relationship network among the 17 subcategories generated through open coding, considering relationships such as causality, parallelism, situational connections, and typology. Consequently, four main categories were deduced: Perceived Psychological Engagement (A1), Perceived Symbolic and Social Value (A2), Perceived Cultural and Environmental Atmosphere (A3), and Perceived Participation Convenience (A4) (refer to Table 3).

**Table 3:** Main results for spindle coding and selective coding.

Core categories	Main categories	Corresponding categories	Encompassed concepts
Marathon attractiveness	A1 perceived psychological engagement	a1 perceived challenge intensity	Physical endurance demand; Mental toughness requirement; Athletic difficulty perception (3 concepts)
		a2 emotional reward	Emotional gratification; Psychological satisfaction; Positive emotional feedback (3 concepts)
		a3 personal growth	Self-development experience; Psychological empowerment; Capacity-building process (3 concepts)
		a4 goal orientation	Achievement-driven mindset; Personal objective pursuit; Purposeful engagement (3 concepts)
		a5 flow experience	Deep task immersion; Enjoyable performance focus; Optimal engagement state (3 concepts)
	A2 perceived symbolic and social value	a6 symbolic meaning	Event symbolic value; Personal life metaphor; Life milestone significance (3 concepts)
		a7 social interaction	Peer bonding opportunity; Interpersonal connection; Social engagement opportunity; team dynamics (4 concepts)
		a8 identity expression	Self-identity display; Personal values projection; Identity reinforcement (3 concepts)
		a9 community connection	Sense of belonging; Group membership identity; Community solidarity experience (3 concepts)
	A3 perceived cultural and environmental atmosphere	a10 cultural integration	Cultural immersion context; Event-local cultural fusion; Sociocultural interaction (3 concepts)
		a11 natural environment	Scenic physical setting; Natural surroundings enjoyment; Eco-aesthetic value (3 concepts)
		a12 event atmosphere	Event excitement vibe; Stimulating competition mood; Emotional ambiance perception (3 concepts)
		a13 spatial aesthetics	Route visual appeal; Spatial design quality; Environmental beauty evaluation (3 concepts)
	A4 perceived participation convenience	a14 time flexibility	Schedule convenience; Temporal participation freedom; Flexible engagement window (3 concepts)
		a15 cost-efficiency	Financial affordability; Participation value ratio; Perceived economic fairness (3 concepts)
		a16 infrastructure support	On-site facility provision; Organizational support quality; Operational efficiency perception (3 concepts)
		a17 traffic accessibility	Transportation convenience; Location accessibility; Trav0065l effort requirement (3 concepts)

### 5.3 Selective coding and model construction

Selective coding represents the final stage of grounded theory analysis, in which previously identified categories are refined and integrated into a cohesive theoretical framework. In this study, the process focused on synthesizing the four axial categories to identify a unifying conceptual core that captures participants’ shared understanding of marathon attractiveness. Guided by Corbin and Strauss (1990) principle of theoretical integration, the analysis aimed not only to connect categories but also to explain the underlying mechanisms that govern individuals’ evaluative and behavioral responses.

Through iterative comparison and abstraction, the core category “Perceived Attractiveness of Marathon Running” was identified. This construct reflects an overarching motivational schema in which individuals evaluate marathons not merely as physical events but as meaningful experiences embedded within personal narratives, social identities, and structural contexts. The four main dimensions—Perceived Psychological Engagement, Perceived Symbolic and Social Value, Perceived Cultural and Environmental Atmosphere, and Perceived Participation Convenience—collectively constitute the interpretive lens through which participants make sense of their engagement with marathon activities. The conceptual structure and the interrelationships between the core category and the four principal dimensions are detailed in Table 4.

**Table 4:** The structural relationship between core categories and main categories.

Structural relationship	Connotation of structural relationship
Perceived Psychological Engagement **→** Marathon Attractiveness	The dimension of Perceived Psychological Engagement constitutes the emotional and existential foundation of marathon attractiveness. Participants often perceive marathon running as a personally transformative journey, characterized by self-discovery, emotional catharsis, and the pursuit of life challenges. These deeply affective and self-referential experiences enhance the perceived meaningfulness of participation, thereby fostering a sustained psychological connection to the sport. As such, intrinsic gratifications play a central role in shaping long-term engagement by reinforcing the internal value of the activity.
Perceived Symbolic and Social Value **→** Marathon Attractiveness	Perceived Symbolic and Social Value enhances marathon attractiveness by embedding individual participation within broader systems of recognition, identity, and affiliation. Running in a marathon allows individuals to gain social visibility, align with aspirational group identities, and construct narratives of perseverance and achievement. These symbolic associations amplify the perceived social significance of participation and contribute to motivation by situating the activity within culturally and relationally meaningful frameworks.
Perceived Cultural and Environmental Atmosphere **→** Marathon Attractiveness	The Perceived Cultural and Environmental Atmosphere dimension refers to the contextual features that enhance the sensory and experiential appeal of marathon events. Scenic routes, festive atmospheres, and safe, well-organized settings foster positive emotional arousal and reduce psychological resistance to participation. These factors not only create favorable first impressions but also support behavioral consistency by reinforcing the anticipated enjoyment and comfort of the running experience.
Perceived Participation Convenience **→** Marathon Attractiveness	Perceived Participation Convenience influence marathon attractiveness through their impact on perceived behavioral control. When traffic barriers—such as cost, time constraints—are minimized, individuals are more likely to perceive marathon participation as feasible and sustainable. This dimension operates primarily through cognitive pathways, increasing individuals’ confidence in their ability to initiate and maintain engagement, thereby making the behavior more actionable.

### 5.4 Theoretical saturation testing

Theoretical saturation refers to “the point at which gathering more data about a theoretical construct reveals no new properties nor yields any further theoretical insights about the emerging grounded theory” (Bryant and Charmaz, 2007). To evaluate theoretical saturation in this study, we adopted the framework proposed by Hennink et al. (2017), which distinguishes between code saturation (i.e., the point when no new codes emerge) and meaning saturation (i.e., when no new dimensions, insights, or properties are observed within established codes).

A total of 45 in-depth interviews were conducted. The first 35 interviews were used for open and axial coding. By the 35th interview, the coding framework had stabilized, and no new codes were identified, indicating that code saturation had been achieved. To assess meaning saturation, we conducted additional coding on the remaining 10 interviews, focusing on whether new conceptual insights or dimensions emerged within the existing categories. These additional interviews reinforced the existing coding structure without contributing new properties or dimensions. In other words, while they offered richer illustrations of the previously identified themes, they did not yield any further theoretical development, suggesting that meaning saturation had also been reached (Hennink et al., 2017).

Based on these findings, we concluded that theoretical saturation was achieved, satisfying both inductive completeness and conceptual robustness expected in grounded theory research.

## 6 Quantitative method

Self-efficacy refers to an individual’s belief in their capacity to execute specific behaviors required to achieve desired outcomes (Bandura and Wessels, 1997). It is a key psychological construct that influences how people approach goals, tasks, and challenges (Waddington, 2023). High levels of self-efficacy are associated with increased confidence, persistence, and resilience in the face of difficulties (Maddux, 2016). Within the domain of physical activity, self-efficacy has consistently been shown to be a key determinant of both the initiation and maintenance of exercise behaviors, including participation in endurance sports such as marathon running (Horcajo et al., 2022).

Given the physical and psychological demands of marathon participation, perceived attractiveness alone may not be sufficient to drive participation intention. Instead, individuals’ perceptions of marathon attractiveness—across dimensions such as emotional reward, social interaction, and environmental appeal—may enhance their belief in their ability to complete such a challenge. In this context, self-efficacy acts as a mediating mechanism, translating perceptual evaluations into behavioral intentions. That is, when individuals perceive marathons as emotionally rewarding, socially engaging, and trafficly accessible, they are more likely to feel capable of participating. This internalized confidence, in turn, strengthens their intention to participate.

While the qualitative phase of this study inductively identified key perceptual dimensions of marathon attractiveness, the constructs of self-efficacy and participation intention were introduced in the quantitative phase based on established psychological theories. These constructs were not derived directly from interview data but were incorporated to test theoretically grounded pathways that may explain how perceived attractiveness influences behavior. In this regard, the quantitative phase does not contradict the inductive logic of the qualitative phase but rather extends the theoretical scope of the emergent constructs by embedding them within a broader motivational framework.

### 6.1 Questionnaire design

Based on the grounded theory analysis and model construction results, this study developed a structured questionnaire to quantitatively assess the perceived dimensions of marathon attractiveness and their impact on individuals’ intention to participate in marathon events. The questionnaire was designed to ensure conceptual alignment with the 17 subcategories and four core dimensions identified in the qualitative phase, while meeting psychometric standards for empirical validation. The final instrument consisted of four sections: (1) an introductory description of marathon running and a screening item (“Have you ever participated in or considered participating in a marathon?”), (2) demographic information (e.g., gender, age, education level, exercise frequency), (3) perceived dimensions of marathon attractiveness, and (4) psychological mediators and the outcome variable—participation intention.

The scale measuring perceived attractiveness of marathon running was developed around four first-order dimensions: Perceived Psychological Engagement, Perceived Symbolic and Social Value, Perceived Cultural and Environmental Atmosphere, and Perceived Participation Convenience. Each dimension was operationalized by integrating the conceptual meanings of its respective subcategories, with multiple items representing each. Since the items in the referenced research or scales were originally in English and the participants’ native language is Chinese, the back-translation method developed by Brislin (1976) was employed in translating the scale. Previous research suggests that, compared to 5-point and 9-point Likert scales, the 7-point Likert scale generally provides greater reliability and validity in addressing complex psychological variables, and for participants, the 7-point Likert scale reduces the fatigue associated with too many options while maintaining sufficient granularity (Lozano et al., 2008; Weijters et al., 2010; Revilla et al., 2014). Therefore, the questionnaire employed a 7-point Likert scale for measurement, with 1 indicating strong disagreement and seven indicating strong agreement. Prior to the full-scale survey, we conducted a pretest of the questionnaire with 30 participants to evaluate item clarity, response comprehension, and completion time. Based on feedback from this pretest, minor wording adjustments were made to improve readability and eliminate ambiguity. These pretest responses were excluded from the final analysis but contributed to optimizing the questionnaire’s design and validity.

The first dimension, Perceived Psychological Engagement, encapsulated individuals’ inner emotional and cognitive gains from marathon participation, with subdimensions including perceived challenge intensity, emotional reward, personal growth, goal orientation, and flow experience. Sample items included: “Participating in marathon running brings me a strong sense of personal achievement,” “Marathon running helps me relieve stress and feel mentally refreshed,” and “I find a sense of meaning and purpose in running marathons.”

The second dimension, Perceived Symbolic and Social Value, referred to the interpersonal and identity-related meanings embedded in marathon participation. It encompassed subcategories such as symbolic meaning, social interaction, identity expression, and community connection. Representative items were: “Marathon running helps me connect with like-minded people.” “Being a marathon runner earns me respect from others,” and “Participating in marathons strengthens my sense of belonging to the running community.”

The third dimension, Perceived Cultural and Environmental Atmosphere, reflected participants’ evaluation of the situational and sensory qualities of the marathon setting. This included cultural integration, natural environment, event atmosphere, and spatial aesthetics. Typical items were: “I enjoy the unique atmosphere during marathon events.” “The route design and scenic environment of marathons enhance my experience,” and “The overall event organization and services influence my willingness to participate.”

The fourth dimension, Perceived Participation Convenience, focused on pragmatic aspects influencing participation, integrating time flexibility, cost-efficiency, infrastructure support, and traffic accessibility. Sample items included: “The cost of participating in marathons is acceptable for me.” “I can arrange my time and schedule to participate in marathons,” and “The location and transportation options make it convenient for me to attend.”

To assess potential psychological mechanisms, the study also included a scale of exercise-related self-efficacy, capturing participants’ confidence in preparing for and completing marathon events. This scale was adapted from Bandura’s self-efficacy framework and prior sports psychology literature (Feltz and Chase, 1998). Additionally, marathon participation intention was measured using three items based on Liu et al. (2023), such as: “I intend to participate in a marathon within the next year” and “I will actively look for information about upcoming marathon events.”

### 6.2 Quantitative data collection

To comprehensively examine the perceived attractiveness dimensions of marathon running, this study employed a combination of online and offline questionnaire distribution methods. Online surveys were administered via the Chinese professional survey platform Wen Juan Xing. At the same time, offline questionnaires were distributed in public spaces with high foot traffic, such as parks, sports venues, and marathon training zones. The data collection period extended from December 2023 to January 2024. To ensure data reliability and exclude careless responses, two attention-check questions were embedded within the questionnaire. Responses that failed these checks were deemed invalid and excluded from further analysis.

A hybrid sampling strategy combining purposive sampling and snowball sampling was adopted. Given the specificity of the target population—individuals with prior marathon experience or a strong interest in marathon participation—probability sampling alone was insufficient to capture the relevant diversity. Purposive sampling was employed to target experienced runners and regular participants in running-related events. In contrast, snowball sampling enabled the recruitment of additional eligible participants via their social networks, such as running clubs and online communities.

A total of 460 questionnaires were distributed, and 426 valid responses were retained after data cleaning, yielding an effective response rate of 92.61%. The valid respondents came from over 20 provinces and regions across China, including Beijing, Shanghai, Jiangsu, Sichuan, Guangdong, Guangxi, Hunan, Jilin, and Gansu, ensuring a geographically diverse sample. Among the participants, 52.35% were male and 47.65% female. In terms of age distribution, 48.12% were between 18 and 28 years old, 42.25% between 29 and 39 years old, and the remaining 9.63% were above or below these brackets.

Regarding marathon engagement, 37.32% of respondents reported participating in at least one official marathon event within the past 2 years, while 62.68% identified themselves as amateur runners or spectators with a strong interest in marathon culture and participation.

### 6.3 Quantitative data analysis

In this part, the data collected from the survey questionnaire were analyzed by SPSS 26.0 and AMOS 26.0 software. The initial step involved examining the validity and reliability of the survey questionnaire through methods such as the KMO test, Bartlett’s test of sphericity, and exploratory factor analysis to ensure the data’s appropriateness for confirmatory factor analysis. Later, confirmatory factor analysis and path analysis were employed for a more in-depth analysis of the data, assessing model fit and main effects to identify the differing impact paths of various perception dimensions on willingness to participate.

## 7 Quantitative results and discussion

### 7.1 Reliability and validity test

To evaluate the psychometric properties of the measurement instrument, an exploratory factor analysis (EFA) was conducted on 27 items, encompassing four first-order dimensions of perceived marathon attractiveness (comprising 5, 4, 4, and 4 items), self-efficacy (7 items), and participation intention (3 items). The Kaiser–Meyer–Olkin (KMO) measure was 0.851, and Bartlett’s test of sphericity reached statistical significance (*p <* 0.001), indicating the data were suitable for factor extraction. Based on the criterion of eigenvalues >1, six distinct factors were identified: perceived psychological engagement, perceived symbolic and social value, perceived cultural and environmental atmosphere, perceived participation convenience, self-efficacy, and marathon participation intention. These factors jointly accounted for 77.125% of the total variance. All items demonstrated satisfactory factor loadings (> 0.60) without significant cross-loadings, supporting the convergent and discriminant validity of the constructs. Moreover, Cronbach’s alpha coefficients for each dimension exceeded the recommended threshold of 0.70, indicating acceptable internal consistency and overall scale reliability (see Table 5).

**Table 5:** Reliability test results.

Latent variable	Number of items	Cronbach’s α
Perceived psychological engagement	5	0.934
Perceived symbolic and social value	4	0.879
Perceived cultural and environmental atmosphere	4	0.883
Perceived participation convenience	4	0.923
Self-efficacy	7	0.937
Marathon participation willingness	3	0.853

### 7.2 Model fit test

Confirmatory factor analysis (CFA) was performed using AMOS 26.0 to assess the factorial validity of the measurement model. The fit indices demonstrated an acceptable model fit: *χ*^2^ = 842.570, df = 309, *χ*^2^/df = 2.727, RMSEA = 0.064, CFI = 0.937, IFI = 0.937, NFI = 0.904, and TLI = 0.928. These results are consistent with commonly accepted thresholds (i.e., RMSEA < 0.08, CFI/TLI > 0.90), indicating that the hypothesized six-factor structure adequately represents the data. Standardized factor loadings for all items were above 0.60, providing convergent validity. Furthermore, the average variance extracted (AVE) values for all constructs exceeded 0.50, and composite reliability (CR) values were above 0.80. The square roots of the AVEs for each latent variable were also greater than the inter-construct correlations, confirming satisfactory discriminant validity. Overall, the measurement model exhibited good construct validity and reliability (see Tables 6, 7).

**Table 6:** Scale question items, sources, and standardized factor loadings.

Dimensions	Items	Standardized regression weights	*P*	Source
Perceived Psychological engagement	Participating in marathon running brings me a strong sense of personal achievement.	0.890	***	In-depth Interview
	Marathon running helps me relieve stress and feel mentally refreshed.	0.875	***	
	I find a sense of meaning and purpose in running marathons.	0.842	***	
	Marathon participation contributes to my overall personal growth.	0.895	***	
	The sense of accomplishment I get from completing marathon is highly motivating.	0.797	***	
Perceived symbolic and social value	Marathon running helps me connect with like-minded people.	0.852	***	In-depth Interview
	Being a marathon runner earns me respect from others.	0.747	***	
	Participating in marathons strengthens my sense of belonging to the running community.	0.777	***	
	I feel proud to share my marathon experiences with others.	0.838	***	
Perceived cultural and environmental atmosphere	I enjoy the unique atmosphere during marathon events.	0.848	***	In-depth Interview
	The route design and scenic environment of marathons enhance my experience.	0.758	***	
	The overall event organization and services influence my willingness to participate.	0.741	***	
	Marathons held in culturally or historically significant locations attract me.	0.876	***	
Perceived participation convenience	The cost of participating in marathons is acceptable for me.	0.848	***	In-depth Interview
	It is easy for me to register and get access to marathon events.	0.758	***	
	I can arrange my time and schedule to participate in marathons.	0.741	***	
	The location and transportation options make it convenient for me to attend.	0.876	***	
Self-efficacy	I am confident in my ability to complete long-distance running.	0.860	***	Feltz and Chase (1998)
	I can overcome physical fatigue during marathon training or participation.	0.793	***	
	I can remain focused and motivated during the entire race.	0.834	***	
	I can develop and follow a running plan to prepare for marathons.	0.802	***	
	I am confident in my ability to deal with unexpected situations during a marathon.	0.801	***	
	I can improve my performance through consistent practice.	0.851	***	
	I believe that I can successfully achieve my running goals.	0.828	***	
Marathon participation willingness	I intend to participate in a marathon event within the next year.	0.782	***	Liu et al. (2023)
	I will actively look for information about upcoming marathon events.	0.804	***	
	I am willing to invest time and effort to prepare for marathon participation.	0.844	***	

The asterisk * indicates significance (***p < 0.001).

**Table 7:** Differential validity test results of scale.

	M ± SD	PPE	PSS	PCE	PPC	SE	MPW
PPE	4.40 ± 1.17	1.000					
PSS	4.58 ± 0.89	−0.037	1.000				
PCE	4.43 ± 0.93	−0.006	−0.119	1.000			
PPC	4.38 ± 1.14	0.010	0.064	−0.098	1.000		
SE	4.41 ± 1.05	0.091	0.246**	0.054	0.136**	1.000	
MPW	4.83 ± 0.94	0.109*	0.271**	0.098*	0.172**	0.486**	1.000

PPE, Perceived Psychological Engagement; PSS, Perceived Symbolic and Social Value; PCE, Perceived Cultural and Environmental Atmosphere; PPC, Perceived Participation Convenience; SE, Self-efficacy; MPW, Marathon Participation Willingness.

### 7.3 Main effect analysis

This study employed Harman’s single-factor test to assess potential common method bias. The results revealed that the first unrotated factor accounted for 24.340% of the total variance, which is well below the recommended threshold of 50% (Podsakoff et al., 2003). Thus, common method bias is unlikely to pose a serious threat to the validity of the findings.

Confirmatory factor analysis was conducted to evaluate the structural model, and the results demonstrated an acceptable model fit: *χ*^2^ = 842.570, df = 309, *χ*^2^/df = 2.727, RMSEA = 0.064, CFI = 0.937, IFI = 0.937, NFI = 0.904, and TLI = 0.928, satisfying the commonly accepted thresholds (Hu and Bentler, 1998; Hu and Bentler, 1999). The structural path analysis showed that all four perceived dimensions of marathon attractiveness had significant positive effects on participants’ willingness to engage in marathon running. Specifically, the standardized path coefficients were as follows: perceived psychological engagement (*β* = 0.092, *p <* 0.05), perceived symbolic and social value (*β* = 0.139, *p <* 0.01), perceived cultural and environmental atmosphere (*β* = 0.102, *p <* 0.05), and perceived participation convenience (*β* = 0.125, *p <* 0.01). These results confirm the significant predictive power of each dimension.

In comparing the strength of these effects, perceived symbolic and social value emerged as the most influential factor, followed by perceived participation convenience, perceived cultural and environmental atmosphere, and perceived psychological engagement. This hierarchical pattern of influence highlights the theoretical robustness and empirical validity of the perceived attractiveness dimensions developed through grounded theory. It further demonstrates their explanatory power in shaping individuals’ intentions to participate in marathon events (see Table 8).

**Table 8:** Path analysis.

Path	Unstandardized path coefficient	Standardized path coefficient	SE	*t*	*p*
PPE → MPW	0.072	0.092	0.035	2.038	0.042
PSS → MPW	0.134	0.139	0.046	2.891	0.004
PCE → MPW	0.087	0.102	0.04	2.198	0.028
PPC → MPW	0.085	0.125	0.031	2.746	0.006

### 7.4 Mediation effects analysis

Mediation analyses were conducted using the bias-corrected bootstrapping method with 5,000 resamples to assess the mediating role of self-efficacy. Results indicated that self-efficacy significantly and partially mediated the effects of all four perceived dimensions of marathon attractiveness on participants’ intention to engage in marathon running. For each path, the 95% confidence interval did not include zero, confirming the statistical significance of the indirect effects (see Table 9).

**Table 9:** Results of mediating effect test.

Path	C total effect	a	b	ab mediating effect	ab (95%CI)	c’ direct effect	Conclusions
PPE → SE → MPW	0.127	0.13	0.422	0.055	[0.006, 0.103]	0.092	Partial mediation effect
PSS → SE → MPW	0.261	0.243	0.422	0.127	[0.066, 0.187]	0.139	Partial mediation effect
PCE → SE → MPW	0.139	0.112	0.422	0.052	[0.003, 0.115]	0.102	Partial mediation effect
PPC → SE → MPW	0.125	0.108	0.422	0.04	[0.004, 0.071]	0.125	Partial mediation effect

These findings suggest that self-efficacy functions as a critical psychological mechanism linking perceptions of marathon attractiveness to participation intention. The partial nature of the mediation further indicates that both direct and indirect pathways are at work, underscoring the multidimensional influence of perceived attractiveness on behavioral intention.

## 8 Overall discussion

This study employed a sequential exploratory mixed-methods design to examine the psychological mechanisms through which perceived attractiveness of marathon running influences individuals’ intention to participate. Through grounded theory analysis of in-depth interviews, we developed a typology comprising four core dimensions—perceived psychological engagement, perceived symbolic and social value, perceived cultural and environmental atmosphere, and perceived participation convenience. These dimensions emerged inductively from participants’ lived experiences, reflecting nuanced subcomponents such as personal growth, symbolic identity expression, community connection, and situational feasibility. This qualitative phase allowed us to conceptualize perceived attractiveness not merely as a cognitive judgment but as a multifaceted psychological and social construction. Subsequently, the quantitative phase validated these emergent dimensions through structural equation modeling (Leguina, 2015), confirming their significant predictive power on participation intention across a broader sample. Moreover, self-efficacy was found to play a consistent and significant partial mediating role across all four dimensions, offering important insights into how external perceptions are internalized as motivational drivers. The alignment between qualitative insights and quantitative outcomes underscores the methodological robustness of our approach and highlights the value of integrating inductive and deductive reasoning. Overall, the mixed-methods design enabled us to move from exploratory theorization to empirical generalization, yielding both conceptual depth and practical relevance.

Among these dimensions, perceived psychological engagement emerged as a foundational element. The affective and existential gratifications associated with marathon running—such as emotional fulfillment, personal growth, and the pursuit of life challenges—are consistent with both hedonic and eudaimonic motivational theories (Ryan and Deci, 2001). These internal rewards reinforce self-efficacy by validating one’s belief in the personal value and attainability of participation (Locke, 1997). Rather than merely physical exertion, marathon running is experienced as a meaningful life journey, strengthening the psychological readiness to engage.

At the same time, the dimension of perceived symbolic and social value emphasizes how interpersonal recognition, group identity, and role modeling contribute to behavioral motivation. This resonates with the tenets of social identity theory and symbolic interactionism (Hogg, 2016), suggesting that individuals internalize social cues and community-based validation as part of their self-concept. When runners feel seen, celebrated, and supported within their social circles, their belief in their capabilities is reinforced, thereby strengthening their intention to participate. This finding highlights the importance of community dynamics and identity-building in promoting sports engagement.

Perceived cultural and environmental atmosphere, while more externally oriented, also plays a critical psychological role. Scenic routes, stimulating competition mood, and vibrant event atmospheres provide more than just physical comfort—they activate sensory appeal and positive emotional states (Han and Ryu, 2009), lowering psychological resistance and reinforcing behavioral scripts. In this sense, favorable running contexts act as facilitators of self-efficacy by reducing perceived barriers and enhancing perceived behavioral fluency.

Similarly, the dimension of participation convenience addresses the practical affordances that shape perceived control. Elements such as time flexibility, cost-efficiency, and infrastructure support influence not only one’s ability to participate but also one’s perceived feasibility of consistent involvement. These findings are consistent with the theory of planned behavior (Bosnjak et al., 2020), which posits that external conditions shape internal readiness by modifying perceived behavioral control. When resources are accessible and constraints are low, individuals feel more confident in their ability to act.

The consistent significance of self-efficacy across all dimensions confirms its central mediating role in the perception-intention linkage. Bandura’s theory of self-efficacy emphasizes that individuals’ confidence in their capabilities determines whether they initiate, sustain, and succeed in behavioral pursuits (Bandura and Wessels, 1997). Our findings extend this theory by demonstrating how diverse sources of perceived attractiveness—ranging from emotional resonance to social validation and contextual ease—converge to strengthen self-belief. This underscores the need to view self-efficacy not as a static trait but as a dynamic construct shaped by multidimensional cues in one’s physical and social environment.

With the growing popularity of virtual marathons and hybrid participation formats—particularly in the post-pandemic era—the concept of event attractiveness is evolving beyond traditional in-person experiences. Online platforms supporting virtual marathons often offer features that simulate physical races through digital, auditory, and visual elements (Liu et al., 2024). Notably, virtual events not only serve as a medium for physical activity but also foster a sense of community and collective accomplishment (Chen et al., 2023). Although our study primarily focuses on in-person participation, the identified dimensions—such as emotional resonance, perceived value, and logistical convenience—may also play a pivotal role in digital participation contexts. Previous research suggests that digital platforms can enhance participant motivation through mechanisms such as gamification, social comparison, and real-time feedback (Huang et al., 2024; Sjukriana et al., 2025). These insights open up opportunities to extend and adapt our conceptual framework to encompass emerging forms of participation, including virtual and hybrid events.

In summary, this study deepens the understanding of how marathon attractiveness is psychologically constructed and operationalized, offering a nuanced model in which environmental, social, and emotional cues interact with cognitive mediators to drive behavioral intentions. These insights contribute to both theory development and practical intervention in the domain of sport participation promotion (Eime et al., 2013).

### 8.1 Practical implications

The findings of this study offer actionable insights for various stakeholders aiming to promote public participation in marathon running through psychologically grounded strategies. Understanding how perceived attractiveness and self-efficacy jointly shape behavioral intentions enables more targeted, effective, and sustainable interventions across organizational, institutional, and policy domains.

For school-based physical education instructors, the emphasis should be placed on cultivating students’ intrinsic experience and personal meaning associated with running. Rather than focusing solely on physical outcomes or competitive achievements, educators can embed running activities within narratives of personal growth (Ntoumanis and Standage, 2009), self-challenge, and emotional fulfillment. Integrating reflective practices, such as goal-setting and journaling, can help students internalize running as a meaningful experience, which in turn enhances self-efficacy and long-term participation intention.

Marathon organizing bodies and event management associations should actively foster the social and symbolic value of marathon participation. This includes cultivating a supportive and inclusive community atmosphere, celebrating diverse participant identities, and promoting social storytelling through media and digital platforms (Funk and James, 2006). Initiatives such as ambassador programs, peer support networks, and community recognition schemes can strengthen runners’ social identity, validate their efforts, and increase their confidence in participating, especially among first-time or hesitant participants. In terms of the situational and environmental aspects, event organizers and local authorities can collaborate to ensure that the running experience is both aesthetically appealing and physically comfortable. Designing routes that integrate scenic landmarks, green spaces, and culturally meaningful locations can enhance emotional engagement. At the same time, ensuring adequate crowd management, hydration stations, medical support, and weather contingency plans contributes to a psychologically safe and positively anticipated environment, lowering cognitive resistance and supporting participation.

Sports administrative departments and public health policymakers should prioritize improving the accessibility and resource availability surrounding marathon training and participation. Subsidized entry fees, low-cost or free training programs, and improved urban infrastructure for running (e.g., well-maintained running tracks, safety lighting, and public equipment) can reduce practical barriers and elevate perceived behavioral control (Sallis et al., 2012). Furthermore, policy incentives that recognize regular participation or community involvement can serve as long-term motivators by transforming running from an isolated leisure activity into a supported lifestyle practice.

Across all interventions, enhancing self-efficacy must remain a core objective (McAuley and Blissmer, 2000). By recognizing and responding to the multiple perceptual cues that influence confidence—from emotional resonance and social inclusion to participation convenience—stakeholders can help individuals transition from passive interest to active engagement. Cultivating a sense of competence and personal agency is crucial not only for increasing initial participation but also for sustaining long-term commitment to marathon running and broader physical activity.

### 8.2 Conclusion

In conclusion, this study provides a comprehensive, theory-informed, and empirically validated framework for understanding how the multidimensional perceived attractiveness of marathon events influences participation intention through the mediating role of self-efficacy. By employing a sequential exploratory mixed-methods design, we were able to not only uncover the lived meanings participants attach to marathon running but also confirm their structural impact at a population level. These findings offer both conceptual advancement and applied relevance, pointing toward the psychological mechanisms and contextual features that support sustained public engagement in endurance sports. Future research can build on this foundation by exploring cross-cultural differences, longitudinal dynamics, and the evolving role of digital technologies in shaping perceived attractiveness and motivational trajectories.

## 9 Limitations

Although this study contributes to both theory and empirical understanding, we acknowledge several limitations that offer valuable directions for future research. While we employed a mixed-methods design and collected data from different regions of China, the generalizability of our findings to other cultural or demographic contexts remains uncertain. Comparative studies across countries, age groups, or experience levels may help validate and extend the applicability of the proposed framework. Our study focused on four core dimensions of perceived attractiveness. However, as marathon events continue to evolve with technological and societal changes, future research could explore additional factors such as digital participation, environmental sustainability, or hybrid race formats.

Furthermore, although our data were collected in the post–COVID-19 era, the pandemic likely had an impact on participants’ motivations and perceptions of marathon running. Many races were canceled or shifted online, potentially increasing the symbolic and emotional significance of participation while also altering perceptions of risk, safety, and accessibility. Future studies could explicitly examine how pandemic-related factors have reshaped runners’ psychological engagement and perceptions of the attractiveness of large-scale public sporting events. Incorporating contextual variables related to the pandemic or conducting longitudinal comparisons may help assess changes in motivational structures before, during, and after public health crises. Such an extension would deepen our understanding of the interplay between external disruptions and internal motivational processes in shaping sport-related behavior.

Another limitation of this study is that our sample did not include participants of virtual or hybrid marathon events, which have grown substantially since the COVID-19 pandemic. While some scholars have criticized virtual environments for lacking the immersive atmosphere of in-person events, emerging research suggests that virtual races can offer alternative forms of social interaction. By meeting social needs in ways that are often more efficient and far-reaching than offline races, virtual events can foster meaningful social engagement and enrich the running experience (Liu et al., 2024). As a result, the psychological mechanisms underlying participation in such formats may differ from those in traditional settings, particularly in terms of technological familiarity, digital community involvement, and perceived authenticity of the experience. We encourage future research to explore how the dimensions of perceived attractiveness manifest in digital contexts, and how they interact with emerging behavioral norms in post-pandemic sport participation.

## Data Availability

The original contributions presented in the study are included in the article/supplementary material, further inquiries can be directed to the corresponding author.
